# Genome assembly of the winter ant, *Prenolepis imparis*

**DOI:** 10.1093/jhered/esae066

**Published:** 2024-12-09

**Authors:** Elizabeth I Cash, Philip S Ward, Merly Escalona, Ruta Sahasrabudhe, Courtney Miller, Erin Toffelmier, Colin Fairbairn, William Seligmann, H Bradley Shaffer, Neil D Tsutsui

**Affiliations:** Department of Environmental Science, Policy, and Management, University of California–Berkeley, Berkeley, CA, United States; Department of Environmental Engineering Sciences, University of Florida, Gainesville, FL, United States; Department of Entomology and Nematology, University of California–Davis, Davis, CA, United States; Department of Biomolecular Engineering, University of California–Santa Cruz, Santa Cruz, CA, United States; DNA Technologies and Expression Analysis Cores, University of California–Davis, Davis, CA, United States; La Kretz Center for California Conservation Science, Institute of the Environment and Sustainability, University of California–Los Angeles, CA, United States; La Kretz Center for California Conservation Science, Institute of the Environment and Sustainability, University of California–Los Angeles, CA, United States; Department of Ecology & Evolutionary Biology, University of California–Los Angeles, CA, United States; Department of Ecology and Evolutionary Biology, University of California, Santa Cruz–Santa Cruz, CA, United States; Department of Ecology and Evolutionary Biology, University of California, Santa Cruz–Santa Cruz, CA, United States; La Kretz Center for California Conservation Science, Institute of the Environment and Sustainability, University of California–Los Angeles, CA, United States; Department of Ecology & Evolutionary Biology, University of California–Los Angeles, CA, United States; Department of Environmental Science, Policy, and Management, University of California–Berkeley, Berkeley, CA, United States

**Keywords:** California Conservation Genomics Project, climate change, cold adaptation, Formicidae, *Prenolepis*, thermal physiology

## Abstract

The winter ant, *Prenolepis imparis*, is one of the most common, widespread, and conspicuous ant species in North America. *P. imparis* is well adapted to cold climates, and consequently, is often noted as the only active ant species during colder months. This specialized life history makes *P. imparis* a useful model organism for exploring thermal physiology and understanding the potential impacts of a warming climate on insects. Phylogeographic studies have revealed deeply divergent lineages across North America, as well as a single collection of an apparent social parasite in California. In light of its distinctive cold adaptation and recently discovered geographic diversity, a better understanding of the underlying genetic patterns of the winter ant is valuable to future conservation efforts for this species. Here, we present a high-quality genome assembly of *P. imparis* from Santa Clara County, California. This genome assembly consists of 787 scaffolds spanning 327.3 Mb, with contig N50 of 901.9 kb, scaffold N50 of 18.7 Mb, and BUSCO completeness of 96.5%. This genome assembly provides an essential foundation for future studies of the winter ant and will be particularly useful for understanding the genetic basis of thermal adaptation, cold resistance, chemical ecology, and the resilience of organisms in response to a changing climate.

## Introduction

**Fig. 1.**
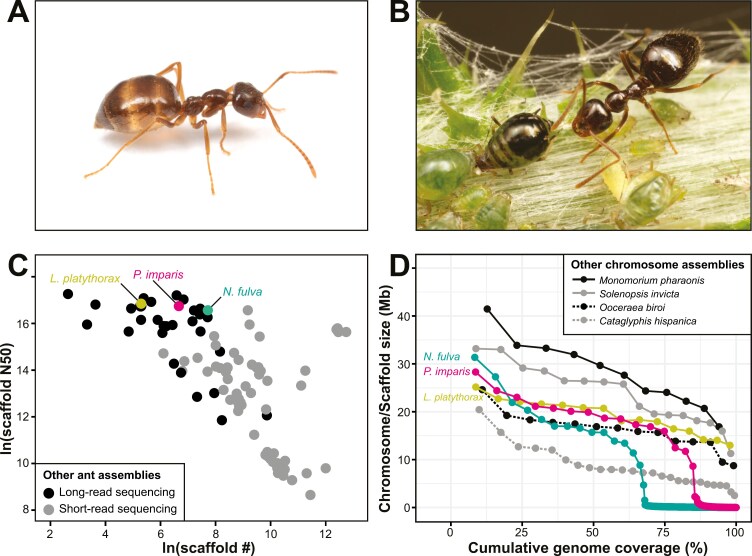
Winter ant, *P. imparis*, and genome assembly comparisons. (A) Representative of *P. imparis* showing a replete worker with nutrient stores in gaster (image credit: Elizabeth I. Cash). (B) Example of a *P. imparis* worker (top right) tending aphid mutualists (bottom left) (image credit: Elizabeth I. Cash). (C) Scatterplot of genome metrics for 97 ant genomes representing 72 species with scaffold-level assemblies accessed via NCBI Datasets (see Supplementary Table S1 for full details). Three focal species, *P. imparis* (this study, long-read sequencing), *L. platythorax* (Feldmeyer et al. 2023, long-read sequencing), and *N. fulva* (Allen et al. 2023, long-read sequencing), are highlighted to compare scaffold assemblies of these closely related taxa with 94 other ant genome assemblies (shaded according to sequencing method, i.e, long- versus short-read lengths). (D) Lineplot of genome metrics comparing 6 ant species with *P. imparis*. The size of each chromosome or scaffold (Mb) is displayed on the *y*-axis and color formatted by species. The cumulative genome coverage (%) is represented on the *x*-axis by the sum of the preceding chromosome or scaffold size(s) up to a given point. Chromosome-level assemblies for 6 ant species (*C. hispanica*, *L. platythorax*, *M. pharaonis*, *N. fulva*, *O. biroi*, and *S. invicta*) are compared with the winter ant, *P. imparis* (this study) showing the similarity between assembled scaffold sizes of *P. imparis* and the chromosome sizes of 2 closely related *Lasiini* species (*L. platythorax* and *N. fulva*; see Supplementary Table S2 for full details).

The winter ant, *Prenolepis imparis*, is a common and widely distributed ant in North America, with a range that extends throughout most of the continental United States and scattered localities in Mexico and from sea level up to 8,000 feet elevation (Wheeler 1930). The species, as currently defined, includes at least 5 highly divergent lineages, plus a possible microgyne social parasite known from a single collection in California (A.L. Wild, personal communication; Tonione et al. 2022). As its name suggests, the winter ant is a cold-adapted species, commonly active during the cooler months of the year or when daytime temperatures are too cold for other ant species. Colonies are often found in cool, shady, and moist microhabitats (Wheeler 1930). Additionally, *P. imparis* exhibits distinctive biological traits such as storing fat and other nutrients in specialized “replete” workers, which act as living energy reserves for the colony during the annual brood-rearing cycle (Tschinkel 1987, Fig. 1A), and the formation of mutualistic relationships with aphids and scale insects (Fig. 1B). These and other features highlight its ecological adaptability and complex social behaviors (Wheeler 1930).

Cold-adapted organisms, including *P. imparis*, are especially vulnerable to a warming climate, and are thus valuable models for understanding how individuals, populations, and species might be affected by climate change. Tests of thermal physiology in *P. imparis* have shown that populations in California vary in their resistance to both heat and cold (Tonione et al. 2020b), and that over 600 genes are differentially expressed in response to heat shock, whereas only 7 genes exhibit significant changes following cold shock (Tonione et al. 2020a). The insights gained from these transcriptomic findings underscore the importance of establishing a reference genome for *P. imparis*, which will serve as a crucial resource for understanding the genetic basis of thermal tolerance and adaptation in the face of climate change.

Here, we report a high-quality de novo genome assembly for *P. imparis* collected in Santa Clara County, California. Existing genomic resources for *P. imparis* include the aforementioned transcriptome sequences and ultraconserved element sequences of specimens from throughout North America (Tonione 2022). Additional genomic resources for closely related species in the tribe *Lasiini* include *Lasius niger*, *Lasius platythorax,* and *Nylanderia fulva*. We highlight comparisons between *P. imparis* (this study) and the whole genome sequences of these 3 *Lasiini* species, as well as the whole genome sequences of other ant species currently available in the NCBI genome database (Fig. 1C and D). As part of the California Conservation Genomics Project (CCGP), this genome assembly will be a foundational resource for broader population genomic studies that will allow us to define taxa, populations, and regions of high conservation concern (Fiedler et al. 2022; Shaffer et al. 2022).

## Methods

### Biological materials

In February 2021, queen *P. imparis* ants were collected during annual nuptial flights in Santa Clara County, California and reared in the lab. To approximate colony-founding conditions, queens were kept individually in water-filled glass tubes stoppered with cotton plugs in ambient temperature conditions (24–26 °C) and total darkness. Queens were monitored once a week for the presence of brood, and male offspring were collected as they developed into pupae or newly eclosed adults. Live samples were flash frozen with liquid nitrogen and stored at –80 °C until processed. DNA for HiFi SMRTbell library construction and sequencing was extracted from 1 adult male *P. imparis* (collection code NDT839.2.m4, NCBI BioSample SAMN35821917) that was produced in captivity by a queen collected from a nuptial flight in Linda Vista Park in Cupertino, Santa Clara County, California (N 37.306842, W –122.060485) on 23 February 2021. DNA for Omni-C library construction and sequencing was extracted from 3 male *P. imparis* pupae (collection codes NDT837.1.m1, NDT837.1.m2, NDT837.1.m3, listed collectively under NCBI Specimen Name NDT837.1, NCBI BioSample SAMN35821884) that were produced in captivity by a single queen collected in February 2021 from Serra Park in Sunnyvale, Santa Clara County, California (N 37.343696, W –122.042545). Given this, the genome assembly was produced from 4 haploid (male) individuals, originating from 2 *P. imparis* populations that were 4.4 km apart.

### High molecular weight genomic DNA preparation

A flash-frozen adult male ant (NDT839.2.m4) was homogenized in 650 µL of homogenization buffer (10 mM Tris-HCL-pH 8.0 and 25 mM EDTA) using TissueRuptor II (Qiagen, Germany; Cat # 9002755). Lysis buffer (650 µL, 10 mM Tris, 25 mM EDTA, 200 mM NaCl, and 1% SDS) and proteinase K (100 µg/mL) were added to the homogenate and incubated overnight at room temperature. Lysate was treated with RNAse A (20 µg/mL) at 37 °C for 30 min and was cleaned with equal volumes of phenol/chloroform using phase-lock gels (Quantabio, Beverly, MA; Cat # 2302830). The DNA was precipitated by adding 0.4 × volume of 5M ammonium acetate and 3 × volume of ice-cold ethanol. The DNA pellet was washed twice with 70% ethanol and resuspended in an elution buffer (10 mM Tris, pH 8.0). DNA yield was 20 ng total as measured by the Qubit 2.0 Fluorometer (Thermo Fisher Scientific, Waltham, MA). Integrity of the high molecular weight genomic DNA (HMW gDNA) was verified on a Femto pulse system (Agilent Technologies, Santa Clara, CA), where 54% of DNA was observed in fragments larger than 50 kb.

The HiFi SMRTbell library was constructed using the SMRTbell gDNA Sample Amplification Kit (Pacific Biosciences [PacBio], Menlo Park, CA; Cat. #101-980-000) and the SMRTbell Express Template Prep Kit 2.0 (PacBio; Cat. #100-938-900) according to the manufacturer’s instructions. High molecular weight genomic DNA was sheared to approximately 10 kb using the Megaruptor 3 system (Diagenode, Belgium; Cat. #B06010003). Sheared genomic DNA was incubated at 37 °C for 15 min to remove single-strand overhangs, followed by DNA damage repair at 37 °C for 30 min, end repair and A-tailing at 20 °C for 30 min and 65 °C for 30 min, and ligation of amplification adapters at 20 °C for 60 min. To prepare for library amplification by PCR, the library was purified with ProNex beads (Promega, Madison, WI; Cat. # NG2002) for 2 PCR amplification conditions at 15 cycles each then another ProNex bead purification. Purified amplified DNA from both reactions was pooled in equal mass quantities for another round of enzymatic steps that included DNA repair, end repair/A-tailing, overhang adapter ligation, and purification with ProNex Beads. The PippinHT system (Sage Science, Beverly, MA; Cat # HPE7510) was used for SMRTbell library size selection to remove fragments < 6 kb. The 10 kb average HiFi SMRTbell library was sequenced at UC Davis DNA Technologies Core (Davis, CA) using 1 8M SMRT cell, Sequel II sequencing chemistry 2.0, and 30-h movies each on a PacBio Sequel II sequencer.

### Omni-C preparation

The Omni-C library was prepared using the Dovetail Omni-C Kit (Dovetail Genomics, Scotts Valley, CA) according to the manufacturer’s protocol with slight modifications. First, specimen tissue (whole individuals, 3 male pupae, specimen numbers NDT837.1.m1, NDT837.1.m2, and NDT837.1.m3) was thoroughly ground with a mortar and pestle while cooled with liquid nitrogen. Subsequently, chromatin was fixed in place in the nucleus. The suspended chromatin solution was then passed through 100 μm and 40 μm cell strainers to remove large debris. Fixed chromatin was digested under various conditions of DNase I until a suitable fragment length distribution of DNA molecules was obtained. Chromatin ends were repaired and ligated to a biotinylated bridge adapter followed by proximity ligation of adapter-containing ends. After proximity ligation, cross-links were reversed and the DNA was purified from proteins. Purified DNA was treated to remove biotin that was not internal to ligated fragments. An NGS library was generated using an NEB Ultra II DNA Library Prep kit (New England Biolabs, Ipswich, MA) with an Illumina compatible y-adaptor. Biotin-containing fragments were then captured using streptavidin beads. The post-capture product was split into 2 replicates prior to PCR enrichment to preserve library complexity with each replicate receiving unique dual indices. The library was sequenced at Vincent J. Coates Genomics Sequencing Lab (Berkeley, CA) on an Illumina NovaSeq 6000 platform (Illumina, CA) to generate approximately 100 million 2 × 150 bp read pairs per GB of genome size.

### Genome assembly

#### Nuclear genome assembly

We assembled the *P. imparis* genome following the CCGP assembly pipeline for haploid genomes, as outlined in Table 1, which lists the tools and non-default parameters used in the assembly. The pipeline uses PacBio HiFi reads and Omni-C data to produce high-quality and highly contiguous genome assemblies. First, we removed the remnant adapter sequences from the PacBio HiFi dataset using HiFiAdapterFilt (Sim et al. 2022) and generated an initial haploid assembly using HiFiasm (Cheng et al. 2022) with the filtered PacBio HiFi reads, specifying no purging and the ploidy corresponding to the sequenced individual, a haploid male. From the generated output, we kept the file corresponding to the primary assembly file. We then aligned the Omni-C data to the assembly following the Arima Genomics Mapping Pipeline (https://github.com/ArimaGenomics/mapping_pipeline) and then scaffolded it with SALSA (Ghurye et al. 2017, 2019).

**Table 1.** Assembly pipeline and software used. Software citations are listed in the text.

Assembly	Software and any non-default options	Version
Filtering PacBio HiFi adapters	HiFiAdapterFilt	Commit 64d1c7b
K-mer counting	Meryl (*k* = 21)	1
Estimation of genome size	GenomeScope	2
De novo assembly (contiging)	HiFiasm (--n-hap 1 -l0)	0.16.1-r375
**Scaffolding**		
Omni-C data alignment	Arima Genomics Mapping Pipeline	Commit 2e74ea4
Omni-C Scaffolding	SALSA (-DNASE, -i 20, -p yes)	2
Gap closing	YAGCloser (-mins 2 -f 20 -mcc 2 -prt 0.25 -eft 0.2 -pld 0.2)	Commit 0e34c3b
**Omni-C** **c** **ontact map generation**		
Short-read alignment	BWA-MEM (-5SP)	0.7.17-r1188
SAM/BAM processing	samtools	1.11
SAM/BAM filtering	pairtools	0.3.0
Pairs indexing	pairix	0.3.7
Matrix generation	cooler	0.8.10
Matrix balancing	hicExplorer (hicCorrectmatrix correct --filterThreshold -2 4)	3.6
Contact map visualization	HiGlass	2.1.11
	PretextMap	0.1.4
	PretextView	0.1.5
	PretextSnapshot	0.0.3
Manual curation tools	Rapid curation pipeline (Wellcome Trust Sanger Institute, Genome Reference Informatics Team)	Commit 4ddca450
**Genome quality assessment**		
Basic assembly metrics	QUAST (--est-ref-size)	5.0.2
Assembly completeness	BUSCO (-m geno, -l hymenoptera)	5.0.0
	Merqury	2020-01-29
**Contamination screening**		
Local alignment tool	BLAST + (-db nt, -outfmt “6 qseqid staxids bitscore std,” -max_target_seqs 1, -max_hsps 1, -evalue 1e-25)	2.10
General contamination screening	BlobToolKit (PacBIo HiFi Coverage, NCBI Taxa ID = 262038, BUSCODB = Hymenoptera)	2.3.3
**Mitochondrial assembly**		
Mitochondrial genome assembly	MitoHiFi (-r, -p 80, -o 1 -a animal) Reference genome: *Lasius spathepus*	2.2

The assembly was manually curated by generating and analyzing its corresponding Omni-C contact maps and breaking scaffolds when misassemblies were identified. We aligned the Omni-C data with BWA-MEM (Li 2013) to generate the contact maps and identified ligation junctions to generate Omni-C pairs (Lee et al. 2022) using pairtools (Open2C et al. 2024). We generated multi-resolution Omni-C matrices with cooler (Abdennur and Mirny 2020) and balanced them with hicExplorer (Ramírez et al. 2018). We used HiGlass (Kerpedjiev et al. 2018) and the PretextSuite (https://github.com/wtsi-hpag/PretextView; https://github.com/wtsi-hpag/PretextMap; and https://github.com/wtsi-hpag/PretextSnapshot) to visualize the contact maps where we identified misassemblies and misjoins. Some of the remaining gaps (joins generated during scaffolding and/or curation) were closed using the PacBio HiFi reads and YAGCloser (https://github.com/merlyescalona/yagcloser). Finally, we checked for contamination using the BlobToolKit Framework (Challis et al. 2020).

#### Genome quality assessment

**Fig. 2.**
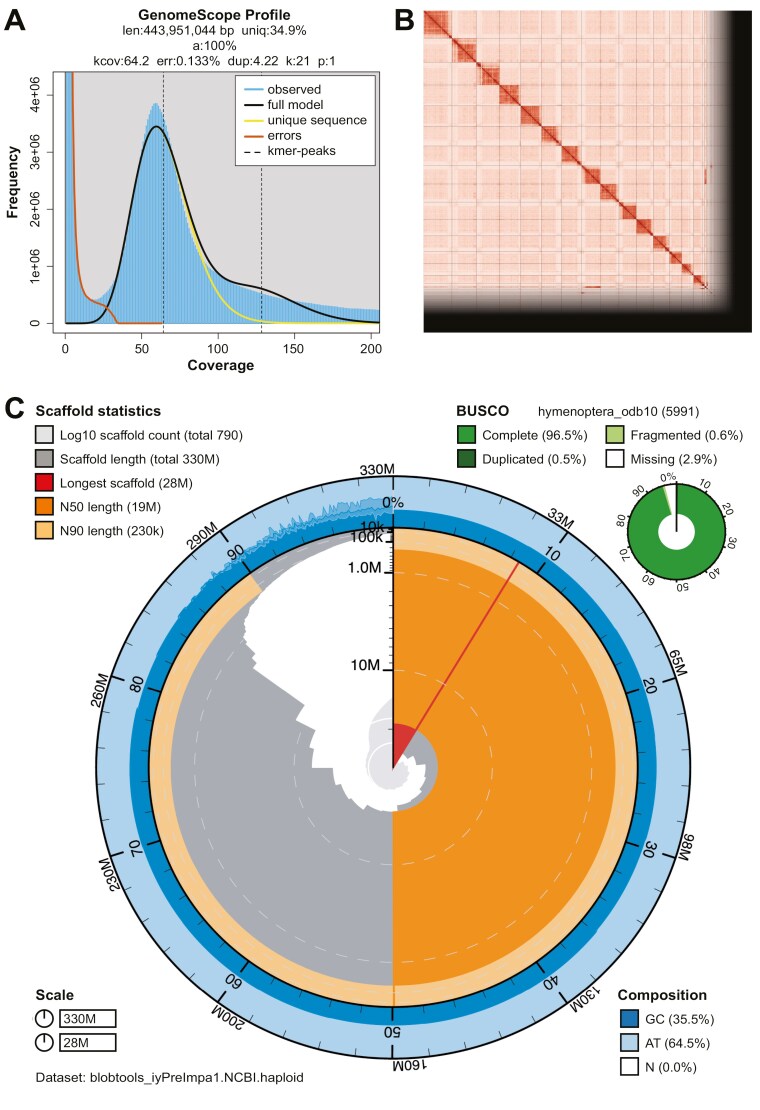
Visual overview of *P. imparis* genome assembly metrics. (A) K-mer spectrum output generated from PacBio HiFi data without adapters using GenomeScope 2.0. The observed unimodal pattern corresponds to a haploid genome. (B) Omni-C contact map for the scaffold-level genome assembly generated with PretextSnapshot. The Omni-C contact map translates the proximity of genomic regions in 3D space to contiguous linear organization. Each cell in the contact map corresponds to sequencing data supporting the linkage (or join) between 2 such regions. Scaffolds are separated by black lines, with higher density corresponding to higher levels of fragmentation. (C) BlobToolKit Snail plot showing a graphical representation of the quality metrics presented in Table 2 for the *P. imparis* assembly (iyPreImpa1) and BUSCO assessment results based on the Hymenoptera set of orthologous genes (*n* = 5,991). The plot circle represents the full size of the assembly. From the inside to the outside, the central plot covers length-related metrics. The red line represents the size of the longest scaffold; all other scaffolds are arranged in size order moving clockwise around the plot and drawn in gray starting from the outside of the central plot. Dark and light orange arcs show the scaffold N50 and scaffold N90 values, respectively. The central light gray spiral shows the cumulative scaffold count with a white line at each order of magnitude. White regions in this area reflect the proportion of Ns in the assembly. The dark versus light blue area around it shows mean, maximum, and minimum GC vs. AT content at 0.1% intervals.

We generated k-mer counts from the PacBio HiFi reads using meryl (https://github.com/marbl/meryl). The k-mer counts were then used in GenomeScope2.0 (Ranallo-Benavidez et al. 2020) to estimate genome features including genome size, heterozygosity, and repeat content. To obtain general contiguity metrics, we ran QUAST (Gurevich et al. 2013). To evaluate genome quality and functional completeness, we used BUSCO (Manni et al. 2021) with both Hymenoptera ortholog databases (hymenoptera_odb10) that contain 5,991 genes. Assessment of base level accuracy (QV) and k-mer completeness was performed using the previously generated meryl database and merqury (Rhie et al. 2020). We further estimated genome assembly accuracy via BUSCO gene set frameshift analysis using the pipeline described in Korlach et al. (2017). Given that the specimen used for the assembly is haploid, measurements of the size of the phased blocks are based on the size of the final contigs. We follow the quality metric nomenclature established by Rhie et al. (2021), with the genome quality code x.y.P.Q.C, where, *x* = log10[contig NG50]; *y* = log10[scaffold NG50]; *P* = log10 [phased block NG50]; *Q* = Phred base accuracy QV (quality value); *C* = % genome represented by the first “*n*” scaffolds, following an estimated karyotype for *Prenolepis* of *n* = 16 inferred from Omni-C contact map results (Fig. 2B) and previous reports of the number of chromosomes for the genus (Goni et al. 1982; Imai et al. 1983).

**Table 2.** Sequencing and assembly statistics, and accession numbers.

**Bio projects & vouchers**						
CCGP NCBI BioProject		PRJNA720569				
Genera NCBI BioProject		PRJNA765835				
Species NCBI BioProject		PRJNA808363				
NCBI BioSample		SAMN35821884, SAMN35821917				
Specimen identification		NDT837.1, NDT839.2m4				
**NCBI genome accessions**						
Assembly accession		GCA_030444845.1				
Genome sequences		JAUDTA000000000				
**Genome sequence**						
PacBio HiFi reads	Run	1 PACBIO_SMRT (Sequel II) run: 3.2M spots, 29.2G-bases, 14.4Gb				
	Accession	SRX21253660				
Omni-C Illumina reads	Run	2 ILLUMINA (Illumina NovaSeq 6000) runs: 54.9M spots, 16.6G-bases, 5.6Gb				
	Accession	SRX21253661, SRX21253662				
**Genome assembly quality metrics**						
Assembly identifier (Quality code^a^)		iyPreImpa1(5.7.P.Q56.C86)				
HiFi Read coverage^b^		65.73X				
Number of contigs		1,480				
Contig N50 (bp)		901,923				
Contig NG50^b^		338,411				
Longest contigs		12,003,945				
Number of scaffolds		787				
Scaffold N50		18,679,404				
Scaffold NG50^b^		16,868,572				
Largest scaffold		28,297,219				
Size of final assembly (bp)		327,284,298				
Phased block NG50^b^		338,411				
Gaps per Gbp (#Gaps)		2117(693)				
Indel QV (Frame shift)		43.84675244				
Base-pair QV		56.5				
k-mer completeness		99.07				
BUSCO completeness^c^ (hymenoptera) *n* = 5991		**C**	**S**	**D**	**F**	**M**
		96.50%	96.10%	0.50%	0.60%	2.80%
Organelles (complete mitochondrial sequence)	Size (bp)	18,821				
	Accession	CM059830				

^a^Assembly quality code x.y.P.Q.C derived notation (from Rhie et al. 2021). *x* = log10[contig NG50]; *y* = log10[scaffold NG50]; *P* = log10 [phased block NG50]; *Q* = Phred base accuracy QV (Quality value); *C* = % genome represented by the first “*n*” scaffolds, following an estimated karyotype for *Prenolepis* of *n* = 16, inferred from Omni-C contact map results (Fig. 2B) and previous reports of the number of chromosomes for the genus (Goni et al. 1982; Imai et al. 1983).

^b^Read coverage and NGx statistics have been calculated based on the estimated genome size of 443.95 Mb.

^c^BUSCO Scores. Complete BUSCOs (C). Complete and single-copy BUSCOs (S). Complete and duplicate BUSCOs (D). Fragmented BUSCOs (F). Missing BUSCOs (M).

#### Mitochondrial genome assembly

We assembled the mitochondrial genome of *P. imparis* from the PacBio HiFi reads using the reference-guided pipeline MitoHiFi (Allio et al. 2020; Uliano-Silva et al. 2023). The mitochondrial sequence of *Lasius spathepus* (NCBI:NC_053901.1) was used as the starting sequence. After completion of the nuclear genome, we searched for matches of the resulting mitochondrial assembly sequence in the nuclear genome assembly using BLAST+ (Camacho et al. 2009) and filtered out contigs and scaffolds from the nuclear genome with a sequence identity >99% and size smaller than the mitochondrial assembly sequence. No other manual curation was performed on the mitochondrial genome.

#### Genome assembly comparisons

We compared basic scaffold-level assembly metrics for 97 ant genomes representing 72 ant species currently available in the NCBI genome database (Supplementary Table S1). Scaffold number versus scaffold N50 (ln transformed) were plotted using ggplot2 in R (Wickham 2016) to visualize differences in contiguity between ant genome assemblies (Fig. 1C). Additionally, scaffold and chromosome sizes (Mb) were plotted relative to genome coverage (%) for 8 ant species including chromosome-level assemblies of 4 non-*Lasiini* species (*Cataglyphis hispanica*, *Monomorium pharaonis*, *Ooceraea biroi,* and *Solenopsis invicta*) and 2 *Lasiini* species (*N. fulva* and *L. platythorax*), as well as scaffold-level assemblies of 2 *Lasiini* species (*L. niger* and *P. imparis* [this study]), to compare mapping results among genome assemblies (Fig. 1D, see Supplementary Table S1 for accession numbers and references, and Supplementary Table S2 for data).

## Results

### Sequencing data

The Omni-C and PacBio HiFi sequencing libraries generated 54.87 million read pairs and 3.17 million reads respectively. The latter yielded 65.73-fold coverage and had an N50 read length 9167 bp; minimum read length 123 bp; mean read length 9181 bp; maximum read length of 33,305 bp (see Supplementary Fig. S1 for read length distribution). K-mer-based analysis of the PacBio HiFi reads with Genomescope 2.0 estimated a genome size of 443.95 Mb and 0.133 % sequencing error. The k-mer spectrum shows an unimodal distribution with a single major peak at ~64 (Fig. 2A).

### Nuclear genome assembly

The size of the final assembly (iyPreImpa1) is similar but not equal to the estimated value from GenomeScope2.0 (Fig. 2A), as has been observed in other taxa (see Pflug et al. (2020) for example). The assembly consists of 787 scaffolds spanning 327.28 Mb with contig N50 of 0.90 Mb, scaffold N50 of 18.68 Mb, longest contig of 12 Mb and largest scaffold of 28.29 Mb. The BUSCO completeness score estimated corresponds to 96.5% using the Hymenoptera gene set, a per base quality (QV) of 56.50, a k-mer completeness of 99.07%, and a frameshift indel QV of 43.84.

During manual curation, we made 359 joins and 24 breaks. In the gap-closing step, we were able to close a total of 8 gaps. We filtered out 2 contigs, 1 corresponding to mitochondrial contamination and 1 corresponding to a mollusc, *Ophicardelus ornatus*. No other contigs were removed. Assembly statistics are reported in Table 2, and its graphical representation is presented in Fig. 2B. We deposited the genome assembly on GenBank (see Table 2 and Data availability section for details).

### Mitochondrial genome assembly

We assembled a mitochondrial genome for *P. imparis* with MitoHiFi. The final mitochondrial sequence has a size of 18,821 bp, with base composition of *A* = 44.43%, *C* = 5.117%, *G* = 10.32%, *T* = 40.14%, and consisting of 21 unique transfer RNAs and 12 protein-coding genes. The mitochondrial genome assembly is available on GenBank (see Table 2 and the Data availability section for details).

### Assembly comparisons

Genome metrics indicate that the winter ant assembly was highly contiguous (787 scaffolds, scaffold N50 of 18.68 Mb), with a scaffold number and scaffold N50 comparable with other available ant genomes generated with long-read sequencing methods (Fig. 1C, Supplementary Table S1). Although chromosome assignments were not determined for *P. imparis*, 16 out of the 787 total scaffolds in the genome assembly have sizes >2 Mb (mean ± SD = 17.50 ± 6.35 Mb), make up >85% of the genome assembly, and are comparable with the average chromosome sizes of genome assemblies from 6 representative ant species (mean ± SD = 16.75 ± 8.42 Mb) including the closely related *Lasiini* species *N. fulva* and *L. platythorax* (Fig. 1D, Supplementary Table S2).

## Discussion

This genome assembly for the winter ant, *P. imparis*, adds to a growing number of genomic resources for the diverse and species-rich ant subfamily Formicinae. *P. imparis*, in particular, is one of the most widespread and recognizable ants in North America, and is particularly abundant during cold seasons, at colder times of the day, and in colder microhabitats. The adaptations that have allowed *P. imparis* to thrive in these thermal and microgeographic niches also make it especially vulnerable to increasing environmental temperatures, as predicted under future climate change scenarios. Thus, with this new genome assembly, *P. imparis* is now poised to become a model system for addressing questions related to the genetic basis of thermal adaptation, tolerance of extreme temperatures, and responses to climate change in an important insect clade.

The *P. imparis* genome assembly presented here has excellent coverage (65.73×) and a high level of BUSCO completeness (96.5%, compared with Hymenoptera, Table 2). Compared with other ant genome assemblies, this winter ant genome is highly contiguous with scaffold and scaffold N50 values similar to that of the chromosome-level genome assemblies of 2 closely related *Lasiini* species, *L. platythorax* and *N. fulva,* as well as other ant species sequenced with long-read methods (Fig. 1C, Supplementary Table S1). The 16 largest *P. imparis* scaffolds comprise 85.5% of the genome assembly and are similar to the assembled chromosome sizes of *L. platythorax* (*n* = 15, Feldmeyer et al. 2023), *N. fulva* (*n* = 16, Allen et al. 2023), and 4 representative ant species (Fig. 1D, Supplementary Table S2). Notably, however, the chromosome number suggested by the assembly of California *P. imparis* (*n* = 16) is double the previously described chromosome number of *Prenolepis nitens* collected in Switzerland (2*n* = 16, *n* = 8, Hauschteck 1962; note that European *P. imparis* was revised to *P. nitens* by Williams and LaPolla [2016]). It is unclear if this difference represents interspecific karyotypic variation between the European and North American species or misreporting of a haploid specimen from Europe as diploid. Studies of another *Prenolepis* species, *Prenolepis jerdoni*, also support a larger haploid chromosome number (*n* = 16–27, Goni et al. 1982; Imai et al. 1984), which is similar to the chromosome number suggested by the assembly results of California *P. imparis*. Taken together, these results indicate that this *P. imparis* genome assembly not only provides a high-quality resource for studying the genetics and evolution of this species but also suggests significant chromosomal variation within the genus, warranting further cytogenetic and genomic investigations.

The assembled size of the *P. imparis* genome (327.3 Mb) is larger than previous genome size estimates by flow cytometry of samples from Orange County, California (4 individuals; 296.2 ± 2.2 Mb; Tsutsui et al. 2008). Genome assemblies for other ants in the *Lasiini* tribe include the tawny crazy ant, *N. fulva* (375.2 Mb, Allen et al. 2023), which has a larger genome than *P. imparis*, and 3 *Lasius* species including the jet black ant, *Lasius fuliginosus* (256.2 Mb, NCBI: GCA_949152495.1), black garden ant, *L. niger* (236.3 Mb, Koronov et al. 2017), and *L. platythorax* (235.8 Mb, Feldmeyer et al. 2023) all of which have notably smaller genomes than *P. imparis*. These differences in genome size highlight the unique genomic characteristics of *P. imparis* within the *Lasiini* tribe, underscoring the importance of this new genome assembly for studies of species-specific traits and genome evolution.

The genome assembly of the winter ant, *P. imparis*, will be a valuable resource for insect conservation, particularly for understanding the response of cold-adapted insects to a warming climate. Moreover, because *P. imparis* exhibits a continental-scale range, from the Pacific coast to the Atlantic Ocean, it is a particularly appealing model for understanding phylogeography, population differentiation, local adaptation, and dispersal. This genome sequence will also help guide our understanding of *Prenolepis* populations in California, which include an extremely rare social parasite, will clarify the taxonomy of the species in this genus, and will contribute to larger goals of the CCGP (Shaffer et al. 2022; Toffelmier et al. 2022). Future population genomic studies that include samples from a broader geographic distribution, including a variety of different climates, elevations, and latitudes, will provide insights into the genetic mechanisms underlying thermal adaptation. This information will, in turn, inform future effective conservation and management strategies for this and other species as they contend with a changing climate (Fiedler et al. 2022).

## Supplementary material

Supplementary material can be found at http://www.jhered.oxfordjournals.org/.

esae066_suppl_Supplementary_Table_S1

esae066_suppl_Supplementary_Table_S2

esae066_suppl_Supplementary_Figure

## Data Availability

Data generated for this study are available under NCBI BioProject PRJNA808364. Raw sequencing data for samples NDT837.1 and NDT839.2.m4 (NCBI BioSamples SAMN35821884 and SAMN35821917) are deposited in the NCBI Short Read Archive under SRR25523627 and SRR25523628 for Omni-C Illumina sequencing data, and SRR25523629 for the PacBio HiFi sequencing data. GenBank accession the assembly is GCA_030444845.1; and for genome sequences JAUDTA000000000. The GenBank accession for the mitochondrial sequence is CM059830.1. Assembly pipeline, scripts, and other data for the analyses presented can be found at the following GitHub repository: www.github.com/ccgproject/ccgp_assembly.
