# Supplementary material for: Genome assembly of the winter ant, Prenolepis imparis
Source: J Hered. 2024 Dec 9;116(3):354–62. doi: 10.1093/jhered/esae066 (PMC12130434; doi:10.1093/jhered/esae066)
Supplement: esae066_suppl_Supplementary_Figure [file esae066_suppl_supplementary_figure.pdf]

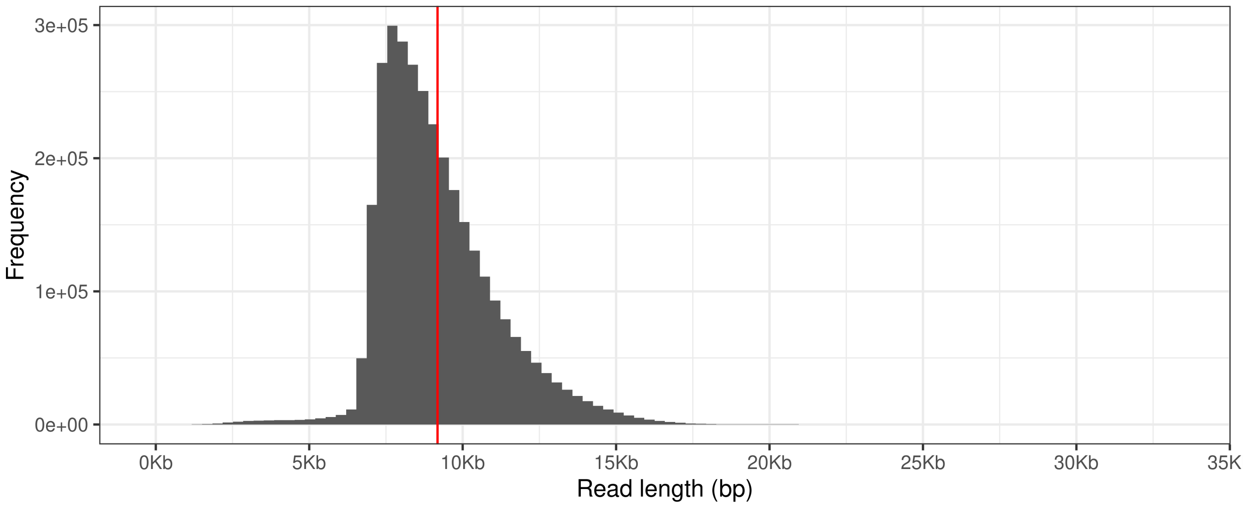

Figure S1. PacBio HiFi sequencing read length distribution of the winter ant, *Prenolepis imparis*, genome.
